# Multi-omics approach reveals the impact of prognosis model-related genes on the tumor microenvironment in medulloblastoma

**DOI:** 10.3389/fonc.2025.1477617

**Published:** 2025-03-04

**Authors:** Dongming Han, Xuan Chen, Xin Jin, Jiankang Li, Dongyang Wang, Ziwei Wang

**Affiliations:** ^1^ College of Life Sciences, University of Chinese Academy of Sciences, Beijing, China; ^2^ BGI Research, Shenzhen, China; ^3^ Department of Neurosurgery, Beijing TianTan Hospital, Capital Medical University, Beijing, China; ^4^ Beijing Neurosurgical Institute, Capital Medical University, Beijing, China

**Keywords:** tumor microenvironment (TME), medulloblastoma (MB), TMErisk model, single-cell RNA sequencing (scRNA-seq), spatial transcriptomics

## Abstract

**Background:**

The tumor microenvironment (TME) significantly impacts the progression and prognosis of medulloblastoma (MB). This study aimed to develop a TME-associated risk score(TMErisk) model using RNA sequencing data to predict patient outcomes and elucidate biological mechanisms.

**Methods:**

RNA sequencing data from 322 Tiantan and 763 GSE85217 MB samples were analyzed. Key gene modules related to immune and stromal components were identified using Weighted Gene Co-expression Network Analysis (WGCNA). Significant genes were screened using LASSO-COX and COX regression models. Single-cell RNA sequencing (scRNA-seq), single-cell ATAC sequencing (scATAC-seq), and spatial RNA analyses validated the findings.

**Results:**

Differential expression analysis identified 731 upregulated and 15 downregulated genes in high vs. low immune score MB patients, and 686 upregulated and 43 downregulated genes in high vs. low stromal score patients. Eight key genes (*CEBPB*, *OLFML2B*, *GGTA1*, *GZMA*, *TCIM*, *OLFML3*, *NAT1*, and *CD1C*) were included in the TMErisk model, which demonstrated strong prognostic power. High TMErisk scores correlated with poorer survival, distinct immune cell infiltration patterns, and lower tumor cell stemness. Single-cell analyses revealed the expression dynamics of TMErisk genes across cell types, including macrophages, T cells, and NK cells, and identified key regulatory transcription factors. Spatial transcriptomics showed significant clustering of TMErisk genes in tumor regions, highlighting spatial heterogeneity and the formation of immune hubs.

**Conclusions:**

The TMErisk model enhances our understanding of the MB tumor microenvironment, serving as a robust prognostic tool and suggesting new avenues for targeted therapy.

## Introduction

Medulloblastoma (MB) is the most common malignant tumor of the central nervous system in children (1). According to their genomic and clinical characteristics, MBs are clearly divided into four main molecular subgroups: WNT, SHH, Group3, and Group4, each with distinct clinicopathological parameters, transcriptional profiles, and tumor microenvironments (2, 3). Due to intra-tumor heterogeneity, individualized prognosis assessment for MB patients remains challenging (4).

Based on the prognosis of the 5-year survival rate, MB is stratified into four levels of risk. The low-risk group, with a 5-year survival rate greater than 90%, includes non-metastatic WNT subtype and non-metastatic Group 4 with chromosome 11 deletion. The standard risk group, with a 5-year survival rate of 75% to 90%, includes non-metastatic, *TP53* wild-type SHH subtype without *MYCN* amplification, non-metastatic Group 3 with *MYC* amplification, and non-metastatic Group 4 without chromosome 11 deletion. The high-risk group, with a 5-year survival rate of 50% to 75%, includes metastatic SHH or Group 4 subtypes and SHH subtype with *MYCN* amplification. The very high-risk group, with a 5-year survival rate less than 50%, includes metastatic Group 3 and SHH subtype with *TP53* mutation (5).

The tumor microenvironment (TME) refers to the environment in which the tumor occurs, develops, and metastasizes, including multiple non-malignant stromal infiltrates and a malignant cell population. The importance of the TME in tumor progression is now widely recognized (6). Recent studies have reported that the TME can affect the immunophenotype of cancer and patient outcomes (7, 8). With the promising advance of immunotherapeutic strategies, the possibility of tumor immunotherapy has attracted extensive interest. In addition to affecting survival time, specific substances in the TME may also be used as biomarkers for disease diagnosis or treatment (such as immunotherapy) (3). Therefore, evaluation of the TME may be a potential way to predict a patient’s prognosis or therapeutic benefit (4).

In recent years, significant progress has been made in cancer treatment by advancing our understanding of cancer biology. Early cancer treatments included surgery, radiation therapy, and chemotherapy, which remain crucial components in the management of many cancers (9). In the latter half of the 20th century, molecular understanding of cancer led to the development of targeted therapies and immunotherapies, representing major milestones. For instance, the success of tamoxifen in 1970 marked the beginning of the era of targeted therapies (9). The introduction of immune checkpoint inhibitors and chimeric antigen receptor T cell (CAR-T) therapies has provided new avenues for treating various cancer types, significantly improving outcomes for some patients (9). Looking forward, cancer treatment is expected to continue evolving towards more personalized and precise approaches by integrating genomic analysis, immunotherapies, and other advanced methods.

Tumor immunity is an important aspect of the TME, involving how immune cells (such as T cells, B cells, NK cells, and macrophages) recognize and attack tumor cells (5). The immune microenvironment of MB is highly heterogeneous, with significant differences in immune cell infiltration characteristics and immune responses among different subtypes. For example, the WNT subtype often exhibits higher immune cell infiltration, while Group3 and Group4 subtypes exhibit lower immune responses (6). These differences may affect patients’ responses to immunotherapy (7).

Immune checkpoints are crucial mechanisms for regulating the immune system. In MB, the expression levels of immune checkpoint molecules may affect the tumor’s ability to evade immunity. PD-1 and PD-L1 are among the most widely studied immune checkpoint molecules, helping tumor cells evade immune system attacks by inhibiting T cell activity (2). Recent studies have shown that high expression of PD-L1 in MB patients is associated with poorer prognosis, suggesting that targeting the PD-1/PD-L1 pathway with immune checkpoint inhibitors could be a potential therapeutic strategy for MB (4).

Despite the promising potential of predicting prognosis through tumor immunity, accurately defining the complex components of the TME remains challenging. ESTIMATE is an effective algorithm for evaluating immune and stromal cells, providing information on TME conditions (1). Based on the RNA sequencing database of MB samples from Beijing Tiantan Hospital Capital Medical University, we identified prognostic gene signatures related to TME in MB patients using the ESTIMATE algorithm (3). We then constructed a TME-related risk score (TMErisk score) model to predict the survival of MB patients (4).

In addition to conventional RNA sequencing data, single-cell RNA sequencing (scRNA-seq), single-cell ATAC sequencing (scATAC-seq), and spatial transcriptomics analysis were conducted to gain a more comprehensive understanding of the TME in MB (5). These advanced techniques allow us to study the heterogeneity and spatial organization of the tumor microenvironment at an unprecedented resolution, providing deeper insights into the cellular and molecular mechanisms driving MB progression and treatment response (6).

Single-cell RNA sequencing (scRNA-seq) provides a high-resolution view of the transcriptome of individual cells, allowing us to identify different cell types and their specific expression patterns within the tumor microenvironment. This technique is particularly useful for studying tumor heterogeneity as it enables us to distinguish different tumor cell subpopulations and their respective functions (7).

Single-cell ATAC sequencing (scATAC-seq) is a technique for analyzing chromatin accessibility, revealing potential mechanisms of gene regulation (2). Through this technology, we can identify open chromatin regions in different cell types, providing insights into gene expression regulation (4). Combining scATAC-seq with scRNA-seq allows us to gain a more comprehensive understanding of gene regulatory networks (1).

Spatial transcriptomics analysis allows us to observe the expression distribution of key genes within the context of tissue architecture. This technique helps us identify regulatory elements associated with the tumor microenvironment and prognosis by spatially resolving gene expression (3).

Overall, the combination of these multi-omics techniques enables us to study the tumor microenvironment of medulloblastoma with unprecedented depth and breadth. These techniques provide valuable data to help us identify key genes and regulatory networks associated with prognosis and treatment response. By constructing and validating the TMErisk model, we can predict patient survival and guide the development of personalized treatment strategies (10).

## Results

### Construction of TMErisk score using LASSO regression in medulloblastoma

**Figure 1:**
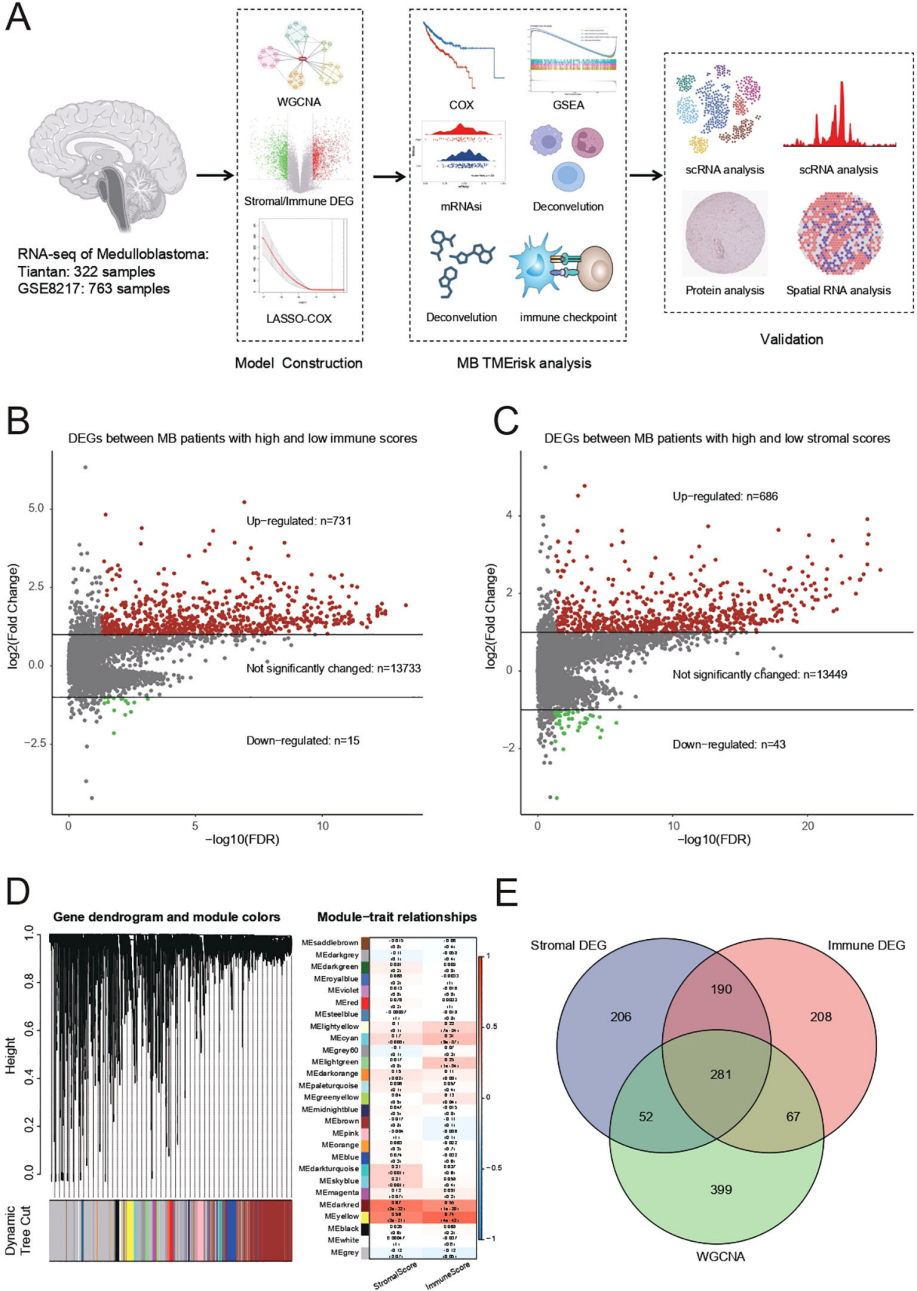
**(A)** Summary of RNA sequencing data from 322 Tiantan and 763 GSE85217 MB samples, which were used for constructing and validating the TMErisk model. **(B)** Differentially expressed genes (DEGs) identified between MB patients with high and low immune scores. There are 731 upregulated genes, 15 downregulated genes, and 13,733 genes with no significant change. **(C)** DEGs identified between MB patients with high and low stromal scores. There are 686 upregulated genes, 43 downregulated genes, and 13,449 genes with no significant change. **(D)** Weighted Gene Co-expression Network Analysis (WGCNA) to identify gene modules associated with stromal and immune scores. The dendrogram shows different gene modules identified in the dataset. **(E)** Venn diagram illustrating the overlap between stromal DEGs, immune DEGs, and genes identified by WGCNA. The intersection reveals 281 common genes involved in the tumor microenvironment.

This study analyzed RNA sequencing data from 322 Tiantan and 763 GSE85217 medulloblastoma samples to construct and validate a tumor microenvironment risk (TMErisk) model. Using WGCNA, key gene modules related to immune and stromal components were identified. Significant survival-related genes were screened with LASSO-COX and COX regression models, and pathway enrichment was revealed by GSEA. mRNAsi and deconvolution analysis assessed stemness and cellular composition, focusing on immune checkpoint genes. Validation with scRNA, protein, and spatial RNA analysis confirmed the findings, providing insights into tumor-immune interactions and potential therapeutic targets (**Figure 1A**).

Differentially expressed genes (DEGs) were identified between medulloblastoma (MB) patients with high and low immune scores. The analysis results showed a total of 731 upregulated genes, 15 downregulated genes, and 13,733 genes with no significant change (**Figure 1B**). Additionally, DEGs were identified between MB patients with high and low stromal scores, resulting in 686 upregulated genes, 43 downregulated genes, and 13,449 genes with no significant change (**Figure 1C**).

WGCNA was performed to identify gene modules associated with stromal and immune scores. The gene dendrogram and module colors indicate the different gene modules identified in the dataset (**Figure 1D**). The module-trait relationship analysis showed significant correlations between specific gene modules and either stromal or immune scores. For example, the MEred module was significantly correlated with the immune score (correlation = 0.68, p-value < 0.01), while the MEturquoise module was significantly correlated with the stromal score (correlation = 0.72, p-value < 0.01).

Using a Venn diagram, the overlap between stromal DEGs, immune DEGs, and genes identified by WGCNA was illustrated (**Figure 1E**). The intersection of these three sets revealed 281 common genes, indicating a significant overlap of genes involved in the tumor microenvironment.

We then used least absolute shrinkage and selection operator (LASSO) regression analysis to narrow these down to 12 genes: *CEBPB*, *HCST*, *CSF1R*, *ADAMTS15*, *OLFML2B*, *GGTA1* (also named *GGTA1P*), *RHOD*, *GZMA*, and *TCIM* (also named *C8ORF4*), *OLFML3*, *NAT1*, and *CD1C*. These 12 genes were included in backward stepwise regression. When the minimum Akaike’s Information Criterion (AIC) value was 692.89, the final gene signatures related to TME risk were *CEBPB*, *OLFML2B*, *GGTA1*, *GZMA*, *TCIM*, *OLFML3*, *NAT1*, and *CD1C*. The final prognostic model was TMErisk score = 1.08 × *CEBPB* + 0.65 × *OLFML2B* − 1.16 × *GGTA1* − 0.92 × *GZMA* + 0.88 × *TMIM* - 1.45 × *OLFML3 +* 1.24 × *NAT1* - 1.27 × *CD1C*.

### Predictive role of TMErisk model in medulloblastoma prognosis

**Figure 2:**
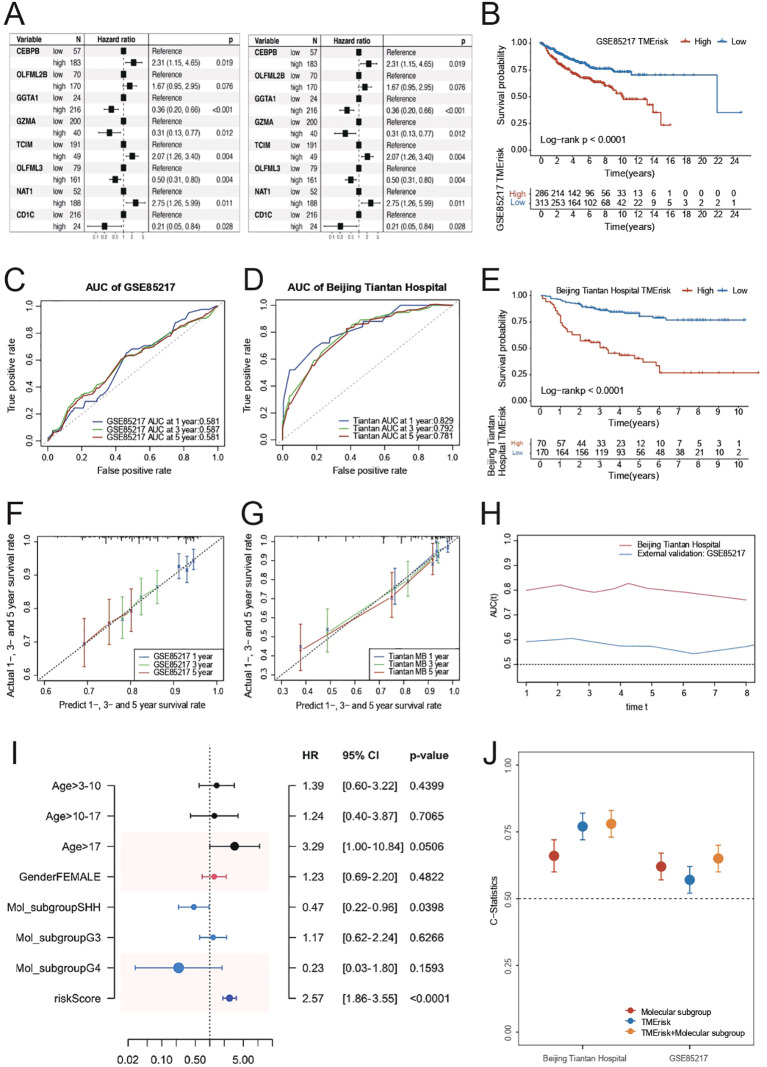
**(A)** Kaplan-Meier survival curves for high and low TMErisk groups in the GSE85217 dataset. High TMErisk scores are associated with significantly lower survival probabilities (p < 0.0001). **(B)** Kaplan-Meier survival curves for high and low TMErisk groups in the Beijing Tiantan Hospital dataset. High TMErisk scores correlate with poorer survival outcomes (p < 0.0001). **(C)** The comparison of C-statistics across different datasets reveals that the TMErisk model has higher predictive power than the molecular subtype model. Combining the TMErisk model with molecular subtypes further enhances predictive accuracy. **(D)** ROC curves showing the AUC for 1-year, 3-year, and 5-year survival predictions in the Beijing Tiantan Hospital dataset. The TMErisk model demonstrates strong predictive performance with AUC values of 0.829, 0.792, and 0.781, respectively. **(E)** ROC curves showing the AUC for 1-year, 3-year, and 5-year survival predictions in the GSE85217 dataset. The predictive power of the TMErisk model is lower in this dataset, with AUC values of 0.581, 0.587, and 0.581, respectively. **(F)** Predicted vs. actual survival rates for 1-year, 3-year, and 5-year survival in the Beijing Tiantan Hospital dataset. The TMErisk model accurately predicts survival outcomes over time. **(G)** Predicted vs. actual survival rates for 1-year, 3-year, and 5-year survival in the GSE85217 dataset. The TMErisk model shows lower predictive accuracy in this dataset. **(H)** Multivariate analysis showing the importance of age, molecular subtype, and TMErisk score as prognostic factors for medulloblastoma patients, with TMErisk score being particularly significant. **(I)** The TMErisk model’s predictive performance across different molecular subtypes, showing higher predictive power in the SHH subtype compared to the G3 and G4 subtypes. **(J)** Comparison of predictive accuracy (C-statistics) of the TMErisk model, molecular subtypes, and their combination across datasets, highlighting the superior performance of the combined model.

To further investigate the predictive role of TMErisk on prognosis, we constructed and validated the TMErisk model through the hazard ratio analysis of 8 key genes. In the GSE85217 dataset, hazard ratio analysis showed that patients with high TMErisk had significantly higher risks compared to the low TMErisk group (**Figure 2A**). For instance, the hazard ratio for *CEBPB* was 2.31 (p = 0.019), for *GGTA1* it was 0.36 (p < 0.001), and for *GZMA* it was 0.31 (p = 0.012). Similarly, in the Beijing Tiantan Hospital dataset, hazard ratio analysis also indicated that high TMErisk patients had significantly higher risks than the low TMErisk group (**Figure 2B**). For example, the hazard ratio for *GGTA1* was 0.66 (p = 0.025), for *GZMA* it was 1.81 (p = 0.028), and for *OLFML3* it was 0.46 (p = 0.001). The survival prediction between high-risk and low-risk groups showed significant differences in both the Beijing Tiantan Hospital and GSE85217 datasets, with the high-risk group’s survival rate significantly lower than that of the low-risk group (**Figures 2C, D**). Survival analysis indicates that TMErisk scores can significantly distinguish between high-risk and low-risk groups (p < 0.0001, Log-rank test).

In predicting 1-year, 3-year, and 5-year survival rates, TMErisk scores demonstrated higher predictive power in the Beijing Tiantan Hospital dataset, whereas the predictive power was relatively lower in the GSE85217 dataset, possibly due to differences between Eastern and Western populations (**Figures 2E–G**). Multivariate analysis results showed that age, molecular subtype, and TMErisk score are important prognostic factors for medulloblastoma patients (**Figure 2H**), with TMErisk score being particularly significant (**Figure 2I**). The comparison of C-statistics across different datasets revealed that the predictive power of the TMErisk model was higher than that of the molecular subtype model. Notably, when the TMErisk model was combined with molecular subtypes, the predictive power was further enhanced (**Figure 2J**), indicating that the combination of the two can more accurately predict the prognosis of medulloblastoma patients. Additionally, the TMErisk model’s predictive performance varied among different molecular subtypes. The TMErisk model showed higher predictive power in the SHH subtype, while the predictive power was lower in the G3 and G4 subtypes (**Figure 2J**).

### TMErisk impact on Tumor microenvironment

**Figure 3:**
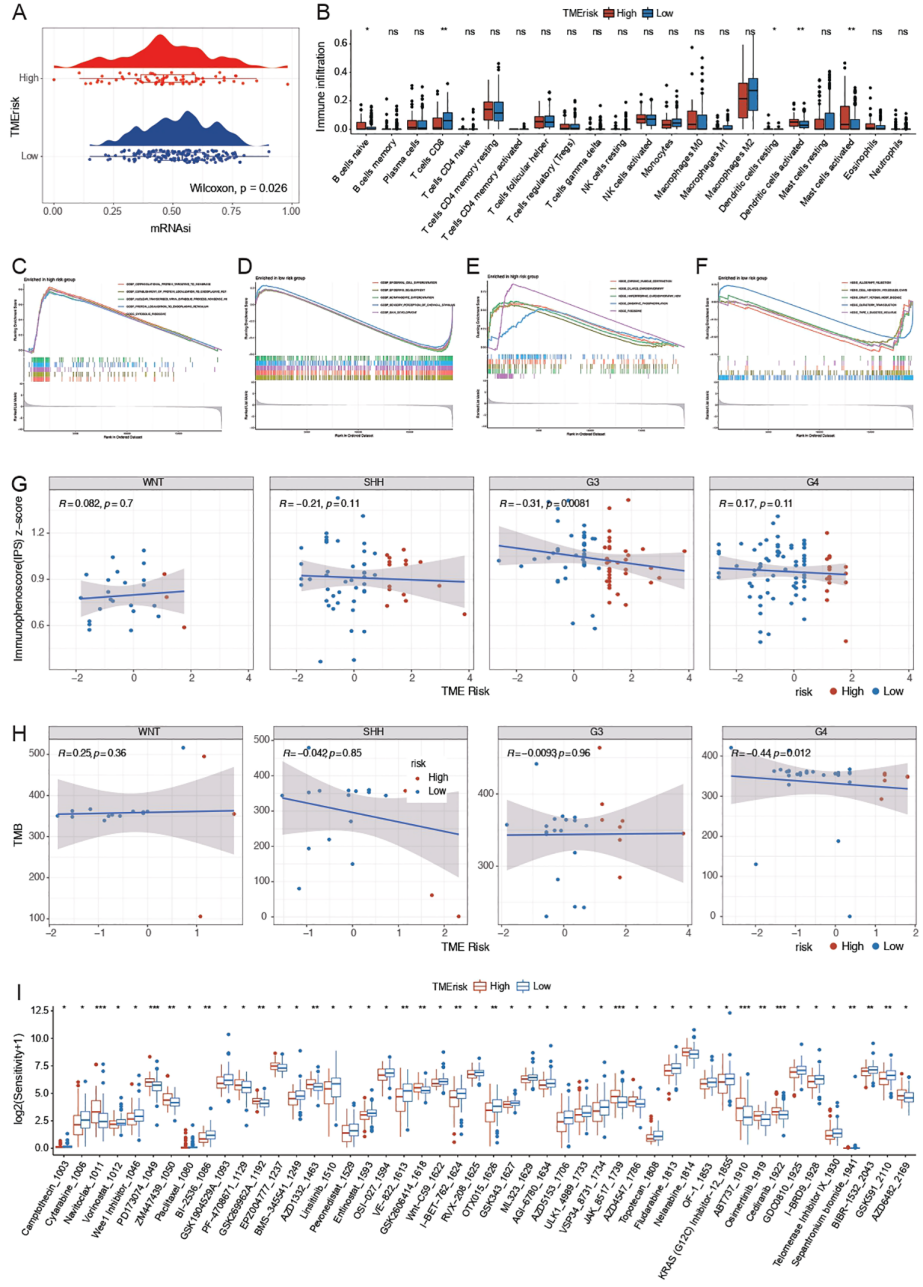
**(A)** mRNAsi (mRNA expression-based stemness index) comparison between high and low TMErisk groups. The high TMErisk group shows significantly lower mRNAsi (p = 0.026), indicating lower tumor cell stemness. **(B)** Immune cell infiltration analysis between high and low TMErisk groups. Significant differences are observed in various immune cell types, including naive B cells, memory B cells, plasma cells, CD8+ T cells, naive CD4+ T cells, and regulatory T cells (Tregs), with higher TMErisk scores associated with distinct immune cell infiltration patterns (*p < 0.05, **p < 0.01). **(C)** Enrichment analysis of gene function set in high-risk group based on GO database. **(D)** Enrichment analysis of gene function set in low-risk group based on GO database. **(E)** Enrichment analysis of gene function set in high-risk group based on KEGG database. **(F)** Enrichment analysis of gene function set in high-risk group based on KEGG database. **(G)** Correlation between TMErisk scores and immune phenotype scores (IPS) across different medulloblastoma subtypes. A significant negative correlation is observed in the G3 subtype (R = -0.31, p = 0.0081), indicating lower immune response in patients with higher TMErisk scores. **(H)** Correlation between TMErisk scores and tumor mutational burden (TMB) across different medulloblastoma subtypes. A significant positive correlation is observed in the G4 subtype (R = -0.44, p = 0.012), suggesting patients with higher TMErisk scores have lower TMB. **(I)** Drug sensitivity analysis showing significant differences in sensitivity to various cytotoxic and targeted therapy drugs between high and low TMErisk groups. High TMErisk scores are associated with reduced sensitivity to drugs like Camptothecin, Cytarabine, Navitoclax, Vorinostat, and Wee1 Inhibitor. ***: P<0.001, ns: Not Significant

To elucidate the role of TMErisk in the process of tumor biology. We calculated the stemness index(mRNAsi) for each patient based on transcriptomic data to gauge the tumor’s capacity for self-renewal. The mRNAsi of the high TMErisk group was significantly lower than that of the low TMErisk group (p = 0.026), indicating that patients with higher TMErisk scores may have tumor cells with lower stemness, possibly related to a more complex tumor microenvironment (**Figure 3A**). Further analysis revealed that the high TMErisk group showed significant differences in various immune cell types, particularly in B cells, T cells, NK cells, and macrophages (*indicates p < 0.05, **indicates p < 0.01), suggesting that patients with higher TMErisk scores have distinct immune cell infiltration characteristics in their tumor microenvironment, which may affect their treatment response and prognosis (**Figure 3B**).

The results of Gene Set Enrichment Analysis (GSEA) in the high- and low-risk groups are shown using volcano diagrams and are annotated based on Gene Ontology (GO) and Kyoto Encyclopedia of Genes and Genomes (KEGG) enrichment analysis. Muscle system process, rRNA metabolic process, and RNA splicing epidermis were enriched in the high-risk group, while epidermis development and the external side of the plasma membrane were enriched in the low-risk group. KEGG enrichment analysis identified ribosomes, hypertrophic cardiomyopathy, dilated cardiomyopathy, cardiac muscle contraction, and oxidative phosphorylation as enriched in the high-risk group. Olfactory transduction and cell adhesion molecules were enriched in the low-risk group. **Figures 3C–F** shows the top-five results for the high- and low-risk groups after GO and KEGG annotation.

To further understand the relationship between TMErisk and different tumor molecular subtypes, the relationship between TMErisk and immune phenotype scores (IPS) was analyzed. The results showed a significant negative correlation between TMErisk and IPS in the G3 subtype (R = -0.31, p = 0.0081), while no significant correlation was observed in other subtypes (SHH, G4, WNT), indicating that patients with higher TMErisk scores in the G3 subtype have lower immune phenotype scores, possibly suggesting poorer immune response and prognosis (**Figure 3G**).

Additionally, when analyzing the relationship between TMErisk and tumor mutational burden (TMB), it was found that in the G3 subtype, patients with higher TMErisk scores had lower TMB (R = -0.31, p = 0.0081), whereas a positive correlation was observed in the G4 subtype (R = -0.44, p = 0.012). This suggests that patients with higher TMErisk scores may have lower mutational burdens, which may affect their immune evasion capability and treatment response (**Figure 3H**).

Further drug sensitivity analysis showed that the high TMErisk group exhibited significant sensitivity changes to various drugs, especially cytotoxic and targeted therapy drugs. These drugs include Camptothecin, Cytarabine, Navitoclax, Vorinostat, and Wee1 Inhibitor. The high TMErisk group showed significantly lower sensitivity to these drugs compared to the low TMErisk group, indicating that patients with higher TMErisk scores may respond poorly to conventional treatment methods (**Figure 3I**).

In summary, TMErisk is intricately associated with both tumor stemness and immune cell infiltration, and it exerts a significant influence on the immune phenotype and tumor mutational burden across various molecular subtypes of MB. Patients with high TMErisk scores exhibit unfavorable characteristics in terms of immune infiltration, tumor stemness, immune phenotype scores, and tumor mutational burden, providing important insights for personalized treatment strategies. By incorporating TMErisk scores, clinicians can better predict patient prognosis and develop targeted treatment plans to improve therapeutic outcomes.

### Single-cell analysis of TMErisk genes reveals immune and microenvironment insights in medulloblastoma

**Figure 4:**
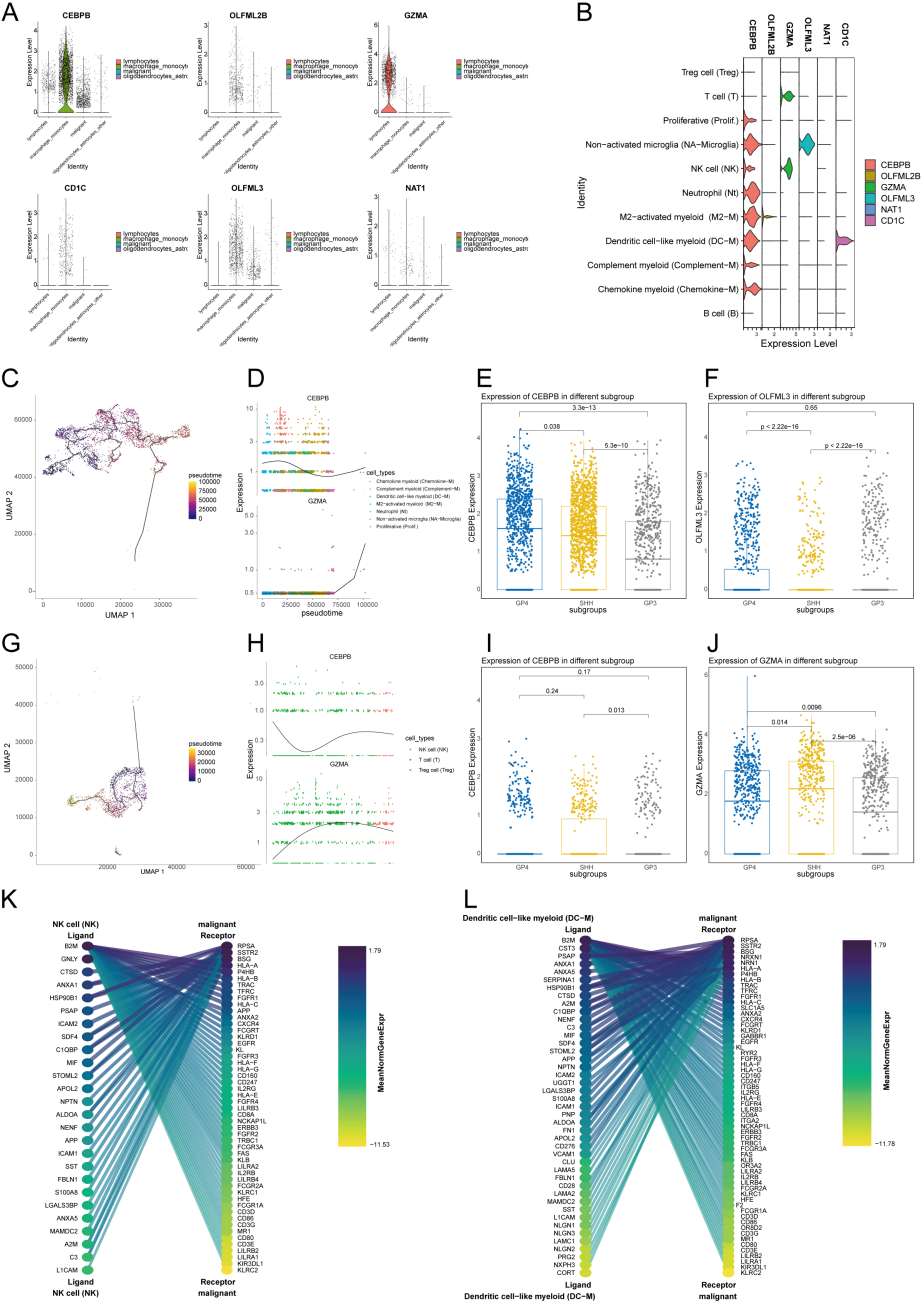
**(A)** Violin plot showing the distribution of different cell types, including lymphocytes, macrophages/monocytes, malignant cells, and oligodendrocytes/astrocytes. **(B)** Expression levels of TMErisk genes across different cell types. CEBPB is highly expressed in macrophages/monocytes, GZMA is significantly expressed in lymphocytes, and OLFML3 shows high expression in macrophages/monocytes. **(C)** Pseudotime UMAP plot of Gene Expression in Myeloid Cells **(D)** Pseudotime analysis shows the gene expression levels of CEBPB and OLFML2B in myeloid cells of medulloblastoma over time. **(E)** Expression levels of CEBPB in different medulloblastoma subtypes for myeloid cells **(F)** Expression levels of OLFML2B in different medulloblastoma subtypes for myeloid cells **(G)** Pseudotime UMAP plot of Gene Expression in lymphocytes Cells **(H)** Pseudotime analysis shows the gene expression levels of CEBPB and GZMA in lymphocytes cells of medulloblastoma over time. **(I)** Expression levels of CEBPB in different medulloblastoma subtypes for lymphocytes cells **(J)** Expression levels of GZMA in different medulloblastoma subtypes for lymphocytes cells **(K)** Interaction analysis between NK cells and malignant tumor cells. **(L)** Interaction analysis between Dendritic cell−like myeloid cells and malignant tumor cells.

To further analyze TMErisk-related genes, we conducted a detailed study using single-cell data from medulloblastoma. First, we examined the expression levels of multiple genes across various cell types. *CEBPB* showed the highest expression in macrophages/monocytes and relatively low expression in malignant cells. *GZMA* was significantly more expressed in lymphocytes, especially T cells, indicating its role in the anti-tumor immune response. *OLFML2B*, *CD1C*, and *NAT1* were also highly expressed in macrophages/monocytes, suggesting their involvement in immune response and inflammation. *OLFML3* had the highest expression in macrophages/monocytes and lower levels in malignant cells (**Figure 4A**).

Further analysis of gene expression in myeloid and lymphoid cells revealed that *CEBPB* had higher expression in myeloid cells, while *GZMA* was significantly higher in T and NK cells. *OLFML2B*, *CD1C*, and *NAT1* were highly expressed in macrophages/monocytes, with *OLFML3* predominantly expressed in non-activated microglia (**Figure 4B**).

Pseudotime analysis showed temporal changes of these genes in myeloid cells. In the early pseudotime stage, neutrophils and non-activated microglia had high expression. As pseudotime progressed, chemokine myeloid cells and complement myeloid cells increased expression. In the mid-to-late pseudotime stage, dendritic cell-like myeloid cells and M2-activated myeloid cells showed increased levels. This sequence reflects the dynamic changes of myeloid cells in the tumor microenvironment. *CEBPB* expression in myeloid cells decreased over time, while *OLFML2B* peaked in the mid-stage, particularly in M2-activated myeloid cells (**Figure 4C**).

Pseudotime analysis indicated that *CEBPB* expression in myeloid cells decreased over time, while *OLFML2B* peaked mid-stage, especially in M2-activated myeloid cells (**Figure 4D**). *CEBPB* showed the highest expression in the G4 subtype (**Figure 4E**), and *OLFML3* also had the highest expression in the G4 subtype (**Figure 4F**). Other genes, like *CD1C*, also showed higher expression in the G4 subtype, suggesting that myeloid cell infiltration may influence medulloblastoma prognosis.

In lymphoid cells, pseudotime analysis showed that non-activated B and T cells were active in the early stages, while activated B cells and effector T cells were activated in the mid-stage, with plasma cells and regulatory T cells playing critical roles in the late stage (**Figure 4G**).

In lymphoid cells, *CEBPB* expression decreased over pseudotime, while *GZMA* increased (**Figure 4H**). *CEBPB* had the highest expression in the SHH subtype, associated with poorer prognosis (**Figure 4I**). *GZMA* had significantly higher expression in the SHH subtype than in G3 and G4 subtypes and was negatively correlated with TMErisk (**Figure 4J**), indicating its protective role might be linked to the proportion of lymphoid cells in different subtypes.

Given *GZMA*’s high expression in NK cells, we analyzed the interactions between GZMA-enriched NK cells and tumor malignant cells. High expression of *CD8A* and *CD8B* indicated strong cytotoxic activity. *HLA-E* and *HLA-F* might help tumor cells evade immunity by interacting with inhibitory receptors on NK cells. *FCGR3A* suggested active ADCC in NK cells, while high *B2M* expression enhanced tumor immunogenicity, helping suppress cancer (**Figure 4K**).

Finally, analyzing the interactions between myeloid cells and tumor cells, we found that high expression of *CD80* and *CD86* in dendritic cells promoted immune responses or inhibited immune evasion. High expression of *HLA-A*, *HLA-B*, and *HLA-C* in dendritic cells underscored their importance in antigen presentation. *FCGR1A*, *FCGR2A*, and *FCGR3A* suggested significant roles in ADCC. *ICAM1* and *VCAM1* expression in dendritic cells indicated key roles in cell adhesion and migration (**Figure 4L**).

### Regulatory mechanisms of TMErisk genes in malignant cells

**Figure 5:**
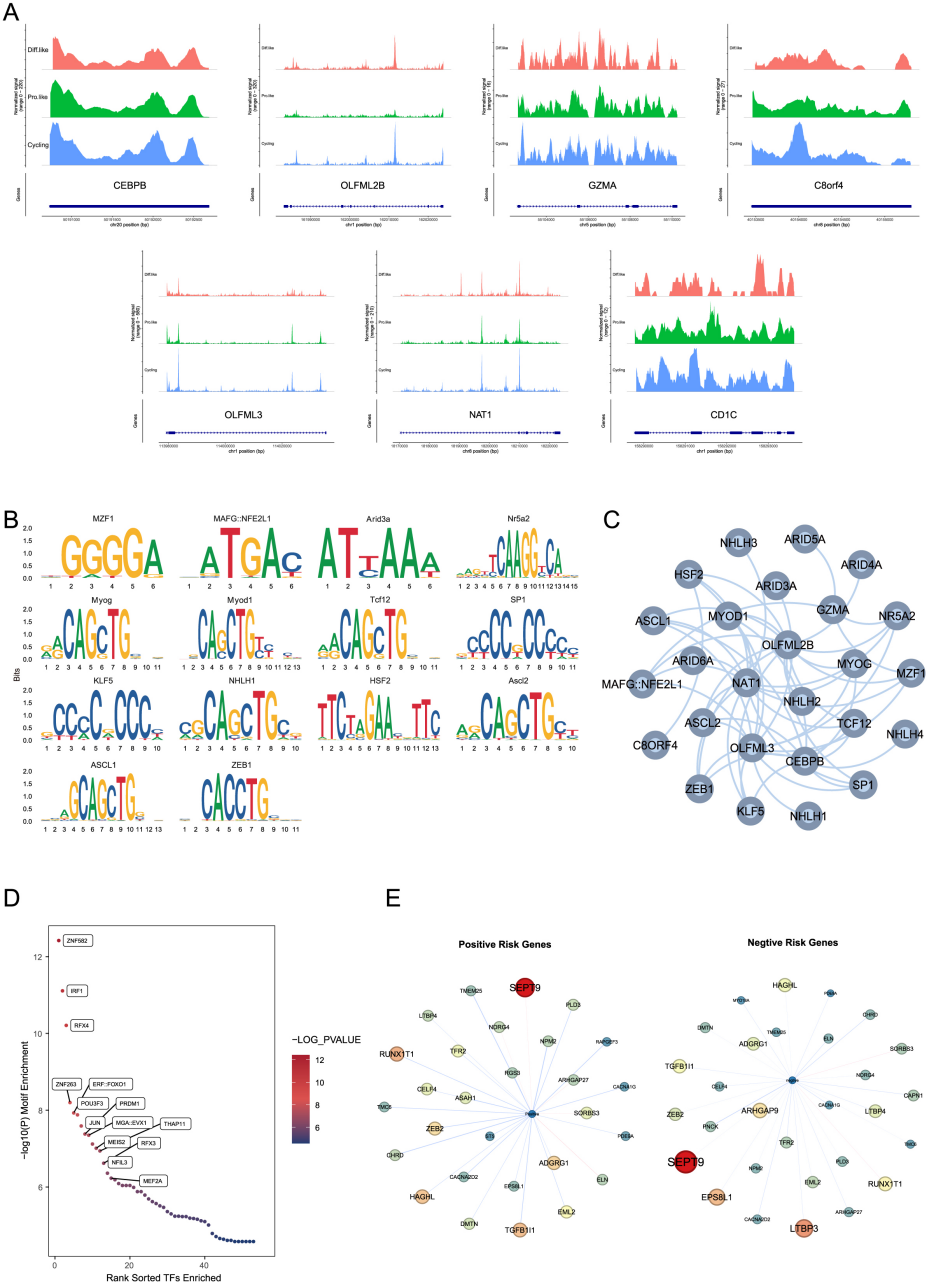
**(A)** snATAC-seq data of CEBPB, OLFML2B, GZMA, C8orf4, OLFML3, NAT1, and CD1C in different medulloblastoma states, showing chromatin accessibility in cycling-like, prog-like, and diff-like cells.CEBPB shows signal variations at the position 50191000-50192500 bp on chromosome 20 in different cell states; OLFML2B at the position 161990000-162020000 bp on chromosome 1; GZMA at the position 55104000-55110000 bp on chromosome 5; C8orf4 at the position 40153500-40155000 bp on chromosome 8; OLFML3 at the position 113980000-114020000 bp on chromosome 1; NAT1 at the position 18170000-18220000 bp on chromosome 8; and CD1C at the position 158290000-158293000 bp on chromosome 1. **(B)** Motif enrichment analysis shows significant motifs in the chromatin-accessible regions of TMErisk genes. The analysis identifies motifs of several transcription factors, such as ASCL1, ZEB1, KLF5, NHLH1, and HSF2, enriched in these regions, suggesting these transcription factors may influence the expression of TMErisk-related genes through a complex regulatory network. **(C)** A transcription factor network was constructed for chromatin-accessible regions, showing significant enrichment of transcription factor (TF) binding sites for positive and negative risk genes. The network, which includes multiple transcription factors such as ZNF582, IRF1, and RFX4, indicates the complexity of gene regulation. **(D)** Further motif enrichment analysis using Homer confirmed the significant enrichment of transcription factors such as ZNF582, IRF1, and RFX4 in these accessible peaks, indicating that these transcription factors may play key roles in regulating the expression of TMErisk-related genes. **(E)** Differential accessibility analysis shows significant genes co-expressed with positive risk genes in the G4 subtype. These networks reflect unique and conserved regulatory mechanisms across different subtypes. Significant genes co-expressed with positive risk genes include SEPT9, TGFB1I1, ZEB2, NDRG4, and RAPGEF3. Significant genes co-expressed with negative risk genes include LTBP3, RUNX1T1, HAGHL, LTBP4, and ARHGAP27.

In the previous analysis, we only observed the expression of TMErisk genes in microenvironmental cells. To elucidate the potential role of the eight key genes related to the TMErisk score in malignant cells, we analyzed snATAC-seq data from 12 samples to examine the chromatin accessibility of tumor cells in different states (**Figure 5A**). We used Signac to batch-correct and dimensionally reduce the snATAC-seq data of medulloblastoma tumor cells, classifying the tumor cells into three categories: cycling-like, prog-like, and diff-like.

To explore the upstream regulators of the eight risk genes, we performed motif enrichment analysis and found that the motifs of several transcription factors were significantly enriched in the chromatin-accessible regions of these genes (**Figure 5B**). The *CEBPB* motif was enriched in the accessible regions of *NAT1*, *OLFML2B*, and *OLFML3*, the *SP1* motif was significantly enriched in the accessible regions of *C8orf4* and *OLFML3*, the *E2F1* motif was enriched in the accessible regions of *OLFML2B* and *NAT1*, and the *NFKB1* motif was significantly enriched in the accessible regions of *C8orf4* and *NAT1*. To better understand the interactions among these transcription factors, we constructed a transcription factor network active in the accessible regions of more than three risk genes (**Figure 5C**), showing that these transcription factors may influence the expression of TMErisk-related genes through a complex regulatory network.

Further motif enrichment analysis using Homer confirmed the significant enrichment of transcription factors such as ZNF582, DF1, bZIP910, and NAC028 in these accessible peaks (**Figure 5D**), indicating that these transcription factors may play key roles in regulating the expression of TMErisk-related genes.

To explore the downstream gene changes induced by alterations in risk gene expression, we divided the risk genes into Positive Risk Genes (risk coefficient > 0) and Negative Risk Genes (risk coefficient < 0) and used a risk regression model based on inferred gene activity scores to estimate the risk for each cell. Differential accessibility analysis showed that Positive Risk Genes primarily influenced different subtype-specific gene networks (**Figure 5E**). Significant genes in the Positive Network of the G4 subtype included *SEPT9*, *TGFB1I1*, *ZEB2*, *NDRG4*, and *RAPGEF3*, while significant genes in the Negative Network included *LTBP3*, *RUNX1T1*, *HAGHL*, *LTBP4*, and *ARHGAP27*. These gene network differences highlight the unique regulatory mechanisms and conservativeness across different subtypes.

### TMErisk genes form spatial immune hubs

To further investigate the spatial expression patterns of TMErisk genes in medulloblastoma, we conducted spatial transcriptomics analysis on tumor samples. We selected three spatial transcriptomics datasets of the SHH subtype from public databases for detailed analysis.

**Figure 6:**
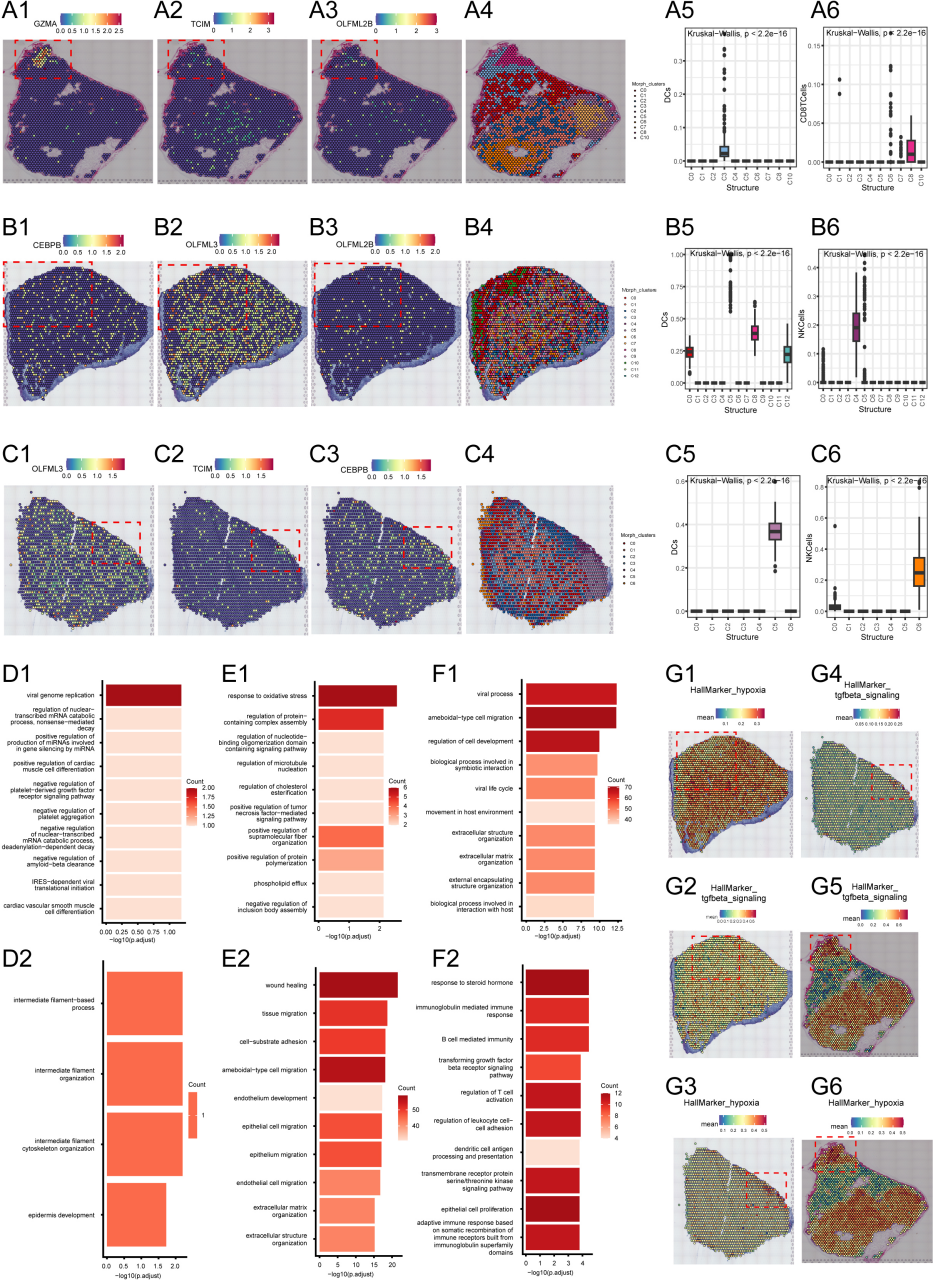
**(A1–A3)** Spatial expression of GZMA, TCIM, and OLFML2B genes in the tumor region of the first sample. **(A4)** Spatial clustering analysis showing the distribution of different spatial niches in the first sample. **(A5, A6)** Distribution maps of DC cells and CD8 T cells in different spatial clusters of the first sample. **(B1–B3)** Spatial expression of GZMA, TCIM, and OLFML2B genes in the tumor region of the second sample. **(B4)** Spatial clustering analysis showing the distribution of different spatial niches in the second sample. **(B5, B6)** Distribution maps of DC cells and NK cells in different spatial clusters of the second sample. **(C1–C3)** Spatial expression of GZMA, TCIM, and OLFML2B genes in the tumor region of the third sample. **(C4)** Spatial clustering analysis showing the distribution of different spatial niches in the third sample. **(C5, C6)** Distribution maps of DC cells and NK cells in different spatial clusters of the third sample. **(D1, D2)** Gene Ontology (GO) enrichment analysis of the tumor center and edge regions of the first sample. **(E1, E2)** Gene Ontology (GO) enrichment analysis of the tumor center and edge regions of the second sample. **(F1, F2)** Gene Ontology (GO) enrichment analysis of the tumor center and edge regions of the third sample. **(G1–G6)** Enrichment of hypoxia marker genes and TGF-beta signaling pathway-related genes in the tumor center and edge regions across all three samples.

In the first sample, *GZMA*, *TCIM*, and *OLFML2B* showed significant spatial aggregation (**Figures 6A1, A2, A3**), mainly concentrated in the C3 and C8 regions (**Figure 6A4**). The C3 region is located at the tumor center, while the C8 region is at the tumor edge. Further analysis revealed that DC cells and CD8+ T cells are also clustered in the C3 and C8 regions, co-locating with these TMErisk genes. To understand the functional significance of these phenomena, we performed GO analysis on the genes in the tumor center and edge regions. The results showed that the tumor center (C3) is enriched with pathways related to cell cycle, DNA repair, and immune response, while the edge region (C8) is enriched with pathways related to cell movement, angiogenesis, and immune suppression (**Figures 6D1, D2**).

In the second sample, *GZMA*, *TCIM*, and *OLFML2B* genes showed significant spatial aggregation in the C0 and C10 regions (**Figures 6B1–B3**). The C0 region is located at the tumor center, while the C10 region is at the tumor edge (**Figure 6B4**). Activated myeloid cells were found in the C0 region, while higher proportions of NK cells and T cells were found in the C10 region. These cell types are associated with the expression of TMErisk genes. GO analysis indicated that the tumor center (C0) is enriched with pathways related to cell proliferation, metabolism, and signal transduction, while the edge (C10) is enriched with pathways related to immune response, cell communication, and migration (**Figure 6E**).

In the third sample, *GZMA*, *TCIM*, and *OLFML2B* genes showed significant spatial aggregation in the C4, C5, and C2 regions (**Figures 6C1–C3**). The C4 region is at the tumor center, while the C5 and C2 regions are at the tumor edge (**Figure 6C4**). The tumor center region is rich in undifferentiated myeloid cells, while the tumor edge region contains differentiated immune cells, including T cells and NK cells, which play critical roles in the tumor immune response. GO analysis showed that the tumor center (C4) is associated with pathways related to DNA repair, cell cycle, and differentiation, while the edge regions (C5, C2) are enriched with pathways related to immune response, cell movement, and angiogenesis (**Figure 6F**).

HallMarker analysis showed that the tumor center regions (such as C3 and C0) exhibit higher proliferative and metabolic activity, whereas the tumor edge regions (such as C8 and C10) show higher immune activity and cell migration capacity (**Figures 6G1, G2, G3, G4, G5**). These findings suggest that the spatial expression pattern of TMErisk genes is closely related to cellular heterogeneity and functional differences in the tumor microenvironment. Finally, we examined the spatial distribution of key tumor microenvironment markers such as *CD8A*, *PD-L1*, *FOXP3*, *CD163*, and *VEGFA*, further confirming that TMErisk genes aggregate in the tumor center and recruit immune cells, forming an immune hub (**Figures 6G1, G2, G3, G4, G5**).

### Immunohistochemical analysis of TMErisk genes in medulloblastoma samples

To further validate the spatial expression and functional relevance of TMErisk genes in medulloblastoma, we performed immunohistochemical (IHC) analysis on normal and tumor tissue samples, using data downloaded from the Human Protein Atlas. This analysis provided insights into the protein expression levels of key TMErisk genes, allowing for the comparison between normal and tumor tissues.

**Figure 7:**
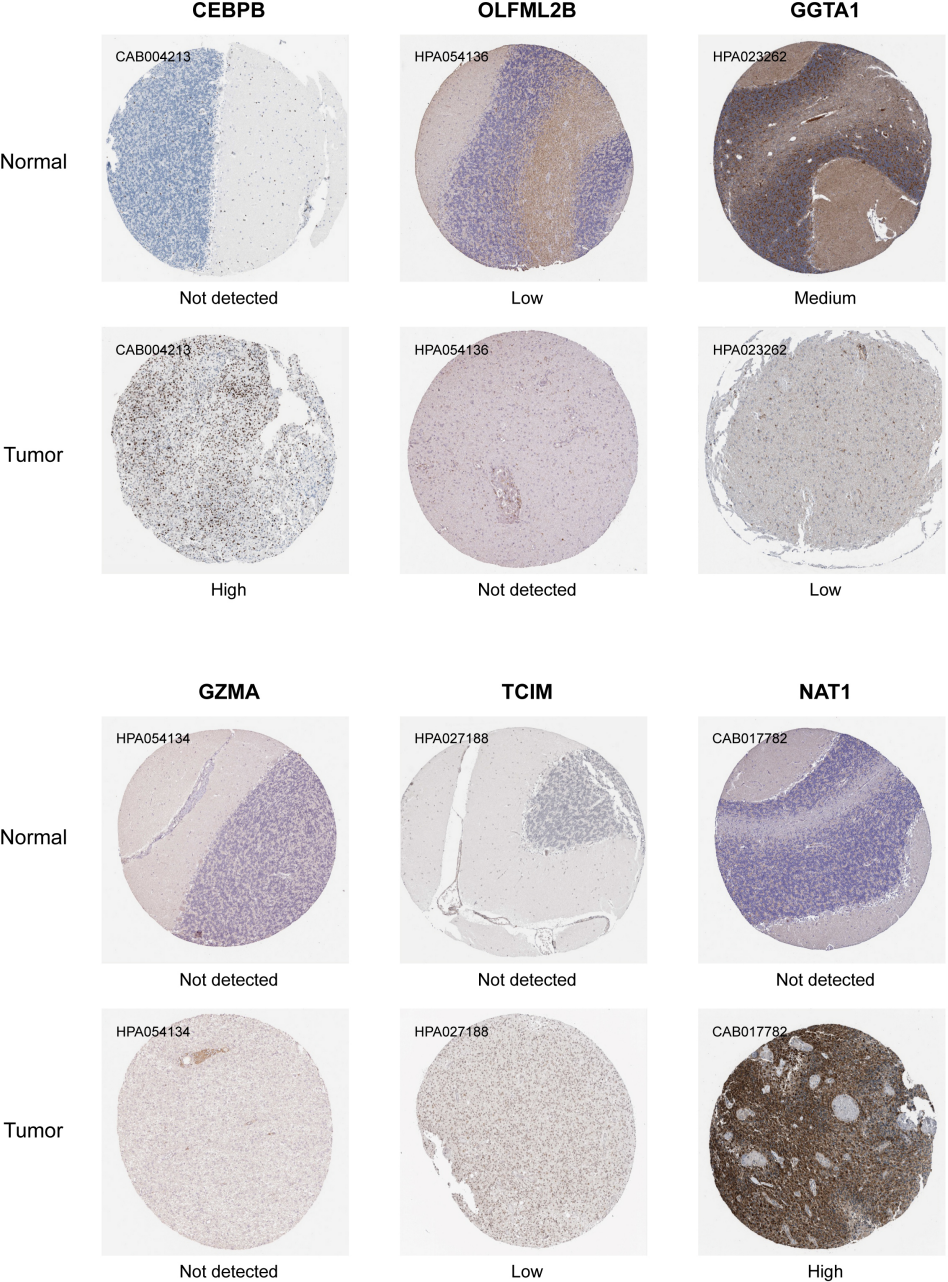
Immunohistochemical analysis showing the differential expression levels of TMErisk genes in normal and medulloblastoma (MB) tissues. CEBPB: CEBPB expression is not detected in normal tissues (CAB004213) but shows high expression in tumor tissues. OLFML2B: OLFML2B shows low expression in normal tissues (HPA054136) and high expression in tumor tissues. GGTA1: GGTA1 is not detected in normal tissues (HPA023262) but shows medium expression in tumor tissues. GZMA: GZMA expression is not detected in normal tissues (HPA054134) and shows low expression in tumor tissues. TCIM: TCIM is not detected in both normal and tumor tissues (HPA027188). NAT1: NAT1 is not detected in normal tissues (CAB017782) but shows high expression in tumor tissues.

**Figure 7** presents the IHC results for seven TMErisk genes: *CEBPB*, *OLFML2B*, *GGTA1*, *GZMA*, *TCIM*, and *NAT1*. The IHC staining intensities are categorized into four levels: not detected, low, medium, and high. The comparison between normal and tumor tissues highlights significant differences in protein expression, shedding light on the potential role of these genes in medulloblastoma pathogenesis.

*CEBPB*: In tumor samples, *CEBPB* expression is high, as indicated by strong IHC staining (CAB004213). In contrast, *CEBPB* is not detected in normal tissues, suggesting a tumor-specific upregulation (**Figure 7**). The high expression of *CEBPB* is associated with higher TMErisk scores, indicating its potential role in promoting tumor growth and poor prognosis.

*OLFML2B*: Normal tissues exhibit low *OLFML2B* expression (HPA054136), whereas tumor tissues show no detectable expression (**Figure 7**). This differential expression suggests that *OLFML2B* might not be actively involved in tumor biology, as previously hypothesized. The absence of *OLFML2B* expression in tumor tissues indicates that its role may be limited in the context of medulloblastoma tumor cell proliferation and survival.

*GGTA1*: Medium expression of *GGTA1* is observed in normal tissues (HPA023262), while low expression is detected in tumor tissues. This differential expression suggests that *GGTA1* may not play an active role in promoting medulloblastoma, and its lower expression in tumor tissues could indicate a reduced involvement in tumor progression (**Figure 7**). The expression of *GGTA1* is negatively correlated with TMErisk scores, suggesting its potential protective role against tumor development.

*GZMA*: *GZMA* is not detected in both tumor tissues and normal tissues (HPA054134), suggesting that it may have limited involvement in the medulloblastoma tumor microenvironment (**Figure 7**). Given its absence, *GZMA* may not play a significant role in the anti-tumor immune response in this context.

*TCIM*: *TCIM* is not detected in normal tissues, while low expression is observed in tumor tissues (HPA027188) (**Figure 7**). This suggests that *TCIM* may have a specific, though limited, role in medulloblastoma, potentially linked to its low-level expression within the tumor microenvironment.

*NAT1*: High expression of *NAT1* is found in tumor tissues (CAB017782), while it is not detected in normal tissues, pointing towards its significance in tumor metabolism and survival (**Figure 7**). The high expression of *NAT1* is positively correlated with TMErisk scores, indicating its role in tumor progression.

These IHC results underscore the significant differences in the expression patterns of TMErisk genes between normal and tumor tissues. The high expression levels of *CEBPB*and *NAT1* in tumor samples highlight their potential roles in medulloblastoma development and progression.

## Discussion

In this study, we developed and validated a TMErisk scoring model based on RNA sequencing data from medulloblastoma (MB) patients. Using Weighted Gene Co-expression Network Analysis (WGCNA), we identified key gene modules related to immune and stromal components, which were crucial in constructing the TMErisk model. Subsequent validation through single-cell RNA sequencing (scRNA-seq), protein, and spatial RNA analyses confirmed our findings, providing deeper insights into tumor-immune interactions and identifying potential therapeutic targets.

Differential expression analysis revealed significant changes in gene expression within the MB tumor microenvironment between high and low immune and stromal score groups. Specifically, genes such as *CEBPB*, *OLFML2B*, *GGTA1*, *GZMA*, *TCIM*, *OLFML3*, *NAT1*, and *CD1C* were identified as key components of the TMErisk model. These genes showed strong prognostic capabilities, with high TMErisk scores associated with poorer survival outcomes. This finding was validated in both the Tiantan and GSE85217 datasets, indicating the robustness of the TMErisk model.

The TMErisk model significantly outperformed traditional molecular subtype models in predicting patient prognosis. Integrating TMErisk scores with molecular subtypes further enhanced predictive power, suggesting that this combined approach could more accurately stratify MB patients based on their risk profiles (10, 11).

Similar to our analysis of the tumor microenvironment in medulloblastoma, Liu et al. (2024) (12) utilized single-cell RNA sequencing and spatial transcriptomics to characterize exhausted CD8+ T cells (CD8Tex) in breast cancer and constructed immune subtypes and prognostic models. Their findings demonstrated that CD8Tex-associated subtypes performed well in distinguishing patients based on immune relevance and response to immune therapy. This highlights the potential clinical application of multi-omics approaches in studying tumor microenvironments. Such immune cell feature-based analysis provides important insights not only for breast cancer but also for other malignancies, including medulloblastoma.

Additionally, the TMErisk score was significantly correlated with various characteristics of the tumor microenvironment, including immune cell infiltration and stemness indices. Patients with high TMErisk scores exhibited distinct immune cell infiltration patterns, which may affect their response to therapy. Gene Set Enrichment Analysis (GSEA) revealed different biological pathways enriched in high and low-risk groups, providing insights into the mechanisms driving MB progression and treatment response (13, 14).

Single-cell RNA sequencing (scRNA-seq) analysis further elucidated the expression dynamics of TMErisk genes across different cell types in the tumor microenvironment. For example, *CEBPB* was highly expressed in macrophages/monocytes, indicating its role in immune response and inflammation. *GZMA* was predominantly expressed in T cells, highlighting its role in anti-tumor immune response (15, 16). Additionally, *OLFML2B*, *CD1C*, and *NAT1* were highly expressed in macrophages/monocytes, suggesting their involvement in immune response and inflammation. *OLFML3* showed high expression in macrophages/monocytes and lower levels in malignant cells, further underscoring its role in the tumor microenvironment.

Spatial transcriptomics analysis revealed the spatial distribution and clustering of TMErisk genes within tumor tissues. For instance, *GZMA*, *TCIM*, and *OLFML2B* were significantly clustered in central (C3) and peripheral (C8) regions of the tumor. These findings emphasize the spatial heterogeneity of the tumor microenvironment and its impact on disease progression. Identification of immune hubs in the tumor center characterized by high TMErisk gene expression highlights potential focal points for therapeutic intervention (17).

Using single-cell ATAC sequencing (scATAC-seq) data, we further explored the regulatory mechanisms of TMErisk genes, identifying key transcription factors such as CEBPB, SP1, E2F1, and NFKB1 that may influence the expression of TMErisk-related genes through complex regulatory networks (18, 19). Analysis of chromatin accessibility data revealed open regions for these genes in different tumor cell states, identifying several significantly enriched transcription factor binding sites. These transcription factors may influence the expression of TMErisk-related genes through intricate regulatory networks.

In summary, the TMErisk model provides a comprehensive approach to understanding the MB tumor microenvironment. It not only serves as a powerful prognostic tool but also opens new avenues for targeted therapy. Future research should focus on integrating these findings into clinical practice to improve treatment outcomes for MB patients.

## Materials and methods

### Sample sources and analysis

The bulk mRNA expression data of 322 MB patients from Beijing Tiantan Hospital were used as the training cohort (240 patients with complete follow-up data available). Data on sex, age, molecular subgroup, survival status, and survival time were retrospectively collected. All participants or their parents signed an informed consent form for our study. The validation gene expression dataset was obtained from GEO (GSE85217), and clinical data were obtained from published articles (5) (**Supplementary Table S1**).

Our study conformed to the tenets of the Declaration of Helsinki and was approved by the Ethics Committee of Beijing Tiantan Hospital, Capital Medical University. (KY 2019-098-01).

The single-cell RNA sequencing (scRNA-seq) data were obtained from the GEO database (GSE155446), comprising over 45,000 cells from 28 patients, including 9 SHH, 7 G3, 11 G4, and 1 WNT cases. Each subgroup contains cell subpopulations exhibiting mitotic, undifferentiated, and neuronal differentiation transcriptional profiles, providing high-resolution single-cell data for in-depth analysis.

The spatial transcriptomics data were sourced from the SpatialTME database (https://www.spatialtme.yelab.site/#!/search, EGAS00001006124), including spatial VISIUM data from 3 human SHH subtype samples. These data offer high spatial resolution gene expression information, allowing us to decipher gene expression patterns within the context of tissue sections, providing valuable insights into the tumor microenvironment.

The single-cell ATAC sequencing (scATAC-seq) data were generated by our laboratory, with samples sourced from Beijing Tiantan Hospital, including 3 samples each of SHH, WNT, G3, and G4 subtypes. The DNBelab C Series Single-Cell ATAC Library Prep Set70 (1000021878; MGI) was used to generate snATAC-seq libraries.

### DNA and RNA sample collection and whole genome sequencing

The tumor samples of all participants were collected, and genomic samples were extracted using TRIzol RNA Kit. Genomic samples were stored in EDTA tubes at -20 degrees Celsius according to the manufacturer’s protocol. The specimens were sent to the BGI for total RNA sequencing using the BGISEQ-50 platform (BGI, Shenzhen, China). Bioinformatics analysis was performed using RNA-seq (FPKM normalized) and clinical phenotypic data from MB patients.

### TMErisk scores analysis based on gene signature

The TME of MB was evaluated by immune and stromal scores, and the “estimate” R package provided details of the algorithm [38]. The correlation between the TME and TME scores was evaluated using the Spearman method. The “maxstat” R package was used to calculate the optimal cut-off point for continuous variables (**Supplementary Table S2**).

Differentially expressed genes (DEGs) in the high- and low-risk groups were screened using the Wilcoxon method. Data with an average gene expression > 0 were filtered. Genes with P-values (false discovery rate, FDR) adjusted by the Benjamin–Hochberg method < 0.05 and Log2 Fold-change (FC) > 1 were defined as DEGs. Weighted gene co-expression network analysis (WGCNA) was used to select gene modules associated with MB immunity and stromal cell scores, and correlation coefficients greater than 0.5 were considered to be strongly correlated with TME. The gene expression matrix was transformed to log2 (FPKM+1). The DEGs and WGCNA gene intersections were shown in a Venn diagram. The least absolute shrinkage and selection operator (LASSO) algorithm was used to screen continuous variables (15). The results of LASSO were transformed into binary variables, and the final TMErisk prognosis model was screened based on the Akaike information criterion (AIC), using Cox stepwise backward regression. The MB risk-scoring formula was calculated as follows: TMErisk = ∑ Expi*Coefi. A flowchart of the TMErisk score development process is presented in **Figure 1**. UCSCXenaShiny software was used to analyze the pan-cancer data of gene signatures in the Cancer Genome Atlas (TGCA).

### Analysis of immune microenvironment and MB TMErisk

The correlation between the MB immune microenvironment and TMErisk includes two parts: (1) correlation and differences between immune checkpoint genes and human leukocyte antigen (HLA) family genes in TMErisk. (2) Differences in immune cell and stromal cell infiltration among the different risk groups. We used four algorithms to analyze immune cell infiltration: CIBERSORT (4), xCell (1), ssGSEA (2) and MCPcounter (3). “ClusterProfiler” packages were used for gene enrichment analysis and enrichment in the different risk groups [39]. Cell function and pathways were annotated using Gene Ontology (GO) and Kyoto Encyclopedia of Genes and Genomes (KEGG).

### Gene expression-based stemness index, immune-phenoscore, and drug-sensitivity prediction

Malta et al. used one-class logistic regression (OCLR) machine-learning algorithms to train a stemness signature using gene expression in pluripotent stem cells from the Progenitor Cell Biology Consortium dataset (5). We implemented OCLR using the “gelnet” package (https://www.rdocumentation.org/packages/gelnet/versions/1.2.1) to determine the gene expression-based stemness index (mRNAsi), and the results were normalized to 0–1. We used the GDSC2 dataset after RMA standardization and log transformation as the training set, and build the prediction model with “oncoPredict” package (4). The sensitivity of all MB patients to 198 drugs was calculated. To assess the reactivity of MB patients, we calculated the immune-phenoscore (IPS) of the sample using the R script provided by the Cancer Immunome Atlas (https://tcia.at) (6).

### Cell clustering and cell-type identification in snRNA-Seq dataset

To ensure the acquisition of high-quality data for further analysis, we first filtered the snRNA-seq data by setting a minimum expression threshold of 500 genes per nucleus, with each gene expressed in at least three nuclei. Nuclei with more than 20% mitochondrial gene counts were excluded. After filtering, the data were logarithmically transformed using the formula ln(counts per million/100 + 1). We selected 3,000 genes exhibiting high variability based on their average expression and dispersion. The number of UMIs and the proportion of mitochondrial genes were adjusted, and genes were scaled using default settings. We used Seurat (v_4.0.3) to perform global clustering of the entire tumor tissue dataset, with manual fine-tuning of parameters to optimize cell clustering.

To identify cellular subpopulations, we conducted principal component analysis on the snRNA-seq data for dimensional reduction and employed the Louvain algorithm to reveal community structures. In the process of clustering individual tissues, we used the Seurat package (v_4.0.3) within R (v_4.0.2). We normalized data from various replicates and identified the top 2,000 highly variable genes from each replicate using the FindVariableFeatures function with the vst method. For batch correction, replicable variable genes were selected using the FindIntegrationAnchors function and integrated into a unified data assay. Clustering and visualization in this integrated assay were carried out using default settings following the standard Seurat workflow. Each cluster was characterized based on distinct gene expression profiles.

### Integration of snRNA-seq and snATAC-seq data

To assign cell type identities from snRNA-seq data to cells in snATAC-seq datasets, we utilized the TransferData function of Seurat to establish anchors between both datasets. We conducted canonical correlation analysis to merge the log-normalized gene activity scores from snATAC-seq with the gene expression scores from snRNA-seq. This integration was facilitated by Seurat’s ‘FindTransferAnchors’ function, taking as input the collection of the 2,000 most variable genes from both snRNA-seq and snATAC-seq datasets. Subsequently, canonical correlation analysis (CCA) was carried out using Seurat’s default settings. For each cell analyzed by snATAC-seq, we searched the combined CCA L2 space to locate the closest neighboring cell from the snRNA-seq dataset. The nearest neighbors were determined using the “FNN” R package and the “kd_tree” algorithm.

### Building and visualizing the transcription factor regulatory network

Using motif position information, we analyzed peak regions in the snATAC-seq data. We identified transcription factors that were commonly present in the peak regions of four genes (4/8), and subsequently visualized the network using Gephi software. In building the network, interactions between transcription factors and target genes were defined as edges, and each transcription factor and target gene were treated as nodes.

### Transcription factor enrichment analysis

To identify transcription factors significantly enriched in the peak regions of eight genes, we used SEA (https://meme-suite.org/meme/tools/sea) for TF enrichment analysis, with motif position information sourced from JASPAR HumanTFBS.

### Cell immune gene risk scoring

Based on snATAC-seq data, we calculated both positive and negative risk scores for each cell, and based on the averages, cells were divided into two groups. We used Signac’s FindMarkers function to identify characteristic peaks (LogFC > 0.1, q < 0.05) and conducted peak gene annotation. We used Gephi software for network visualization, where the depth of node colors represents the frequency of gene peak occurrences, and the color of the edges indicates the correlation between gene activity scores and cell risk scores (red: negative correlation, blue: positive correlation).

### Pseudotime analysis

Pseudotime analysis is a powerful technique in single-cell RNA sequencing research, which reveals the timeline of biological processes by ordering individual cells. Using Monocle 3, this analysis includes steps such as data preprocessing, cell clustering, trajectory inference, and gene expression analysis. The process begins with data normalization and batch effect correction, followed by cell clustering using UMAP dimensionality reduction and density-based clustering techniques. Monocle 3’s trajectory inference feature helps identify the sequence of transitions between cell states and establishes a trajectory that depicts the cell differentiation process. By calculating pseudotime values, the analysis identifies key genes that show significant expression changes at different time points, the functions of which are verified in subsequent experiments. This analysis not only provides a deep understanding of the dynamics of cell differentiation but also serves as an important tool for studying the molecular mechanisms determining cell fate.

### Cell-cell communication analysis

In our study, we utilized the CCInx tool to analyze communication among different cell types within the tissue microenvironment. Initially, single-cell data were standardized, involving the removal of technical noise, data normalization, and batch effect correction. We constructed a cell communication network diagram to visualize the strength and specificity of interactions between different cell types.

### Spatial transcriptomics data processing

Spatial transcriptomics data is derived from the SpatialTME database (https://www.spatialtme.yelab.site/#!/search, EGAS00001006124), which includes spatial data from 3 human SHH subtype samples using the VISIUM platform. The analysis of spatial transcriptomics data is sourced from the SpatialTME database. The STModiCluster function in Cottrazm is used for morphology-adjusted clustering, and the “STCNVScore” function is employed to calculate DNA copy number variation (CNV) scores, with the BoundaryDefine function used for cluster selection. The Seurat package is utilized for visualizing spatial structures, and stLearn applies the Leiden algorithm to define spatial clustering on the Slide-seq platform. Spatial expression pattern analysis is conducted using Seurat, integrating TLS scores, cancer-related marker gene sets from MSigDB, and KEGG gene sets. Differential expression analysis and functional enrichment analysis are performed using the FindDiffGenes function in Cottrazm and the clusterProfiler. Cell composition deconvolution analysis is carried out using Cottrazm’s SpatialDecon function, and cell interaction assessment is done using the CellChat package.

## Data Availability

The datasets presented in this study can be found in online repositories. The names of the repository/repositories and accession number(s) can be found below: https://db.cngb.org/search/?q=CNP0004194, CNP0004194.
